# Facial Affect Recognition Training Through Telepractice: Two Case Studies of Individuals with Chronic Traumatic Brain Injury

**DOI:** 10.5195/IJT.2015.6167

**Published:** 2015-07-29

**Authors:** JOHN WILLIAMSON, EMI ISAKI

**Affiliations:** NORTHERN ARIZONA UNIVERSITY, FLAGSTAFF, AZ, USA

**Keywords:** Facial Affect Recognition Training, telepractice, Theory of Mind, traumatic brain injury

## Abstract

The use of a modified Facial Affect Recognition (FAR) training to identify emotions was investigated with two case studies of adults with moderate to severe chronic (> five years) traumatic brain injury (TBI). The modified FAR training was administered via telepractice to target social communication skills. Therapy consisted of identifying emotions through static facial expressions, personally reflecting on those emotions, and identifying sarcasm and emotions within social stories and role-play. Pre- and post-therapy measures included static facial photos to identify emotion and the Prutting and Kirchner Pragmatic Protocol for social communication. Both participants with chronic TBI showed gains on identifying facial emotions on the static photos.

In the United States, approximately 1.7 million individuals sustain traumatic brain injuries (TBIs) each year (Faul, Wald, Xu, & Coronado, 2010). Following injury, some of these individuals will have long-term challenges with social communication or pragmatics. While individuals with TBI may demonstrate adequate syntactic and lexical capabilities, they are often challenged to maintain personal relationships due to paralinguistic deficits (Angeleri et al., 2008).

## THEORY OF MIND AND/OR EXECUTIVE FUNCTION

Several theories have emerged to explain the cause and nature of pragmatic deficits in individuals with TBIs. One theory posits that deficits in social cognition are the central cause of pragmatic deficits. Social cognition has been defined as “…the ability to process social information” (Byom & Turkstra, 2012, p. 310) and involves a variety of mental processes. One primary aspect of social cognition is Theory of Mind (ToM) which can be explained as “…a person’s ability to form representations of other people’s mental states and to use these representations to understand, predict, and judge utterances and behavior” (Martin & McDonald, 2003, p. 454). This theory supports findings that problems with social cognition relating to ToM may cause pragmatic deficits. Another aspect of social cognition involves the ability to perceive emotions, which can also be difficult for some individuals with TBI. Spikman et al. (2013) found a significant correlation between facial affect recognition and behavior changes in patients with TBI. The researchers gave 51 participants with moderate to severe TBI a test for emotion recognition and a questionnaire for behavioral problems. In particular, impaired emotion recognition, especially with regard to the emotions of sadness and anger, was correlated with behavior concerns and impaired self-awareness. Understandably, a deficit in reading facial expressions would impact an individual’s ability to use ToM in properly making inferences about others and further impair social functioning.

A second theory for the cause of pragmatic problems in individuals with TBI suggests that deficits in executive function are the central underlying contributors of the disorder. Executive function can be defined as “…cognitive control processes that include self-regulation, allocation of attention, maintenance and manipulation of information over time, planning, and task management” (Douglas, 2010, p. 366).

Douglas (2010) compared 43 adult participants with severe TBI and their close relatives to 43 additional adult participants with no neurological issues and their relatives. All individuals with TBI were at least two years post-injury. Evaluations were completed on a range of executive function tasks using the FAS verbal fluency test, the Speed and Capacity of Language Processing test, and the Rey Auditory Verbal Learning Task. Pragmatic function was measured via the La Trobe Communication Questionnaire. Results found that the participants with TBI violated the conversational principles of relation, manner, and quantity, which created frequent and chronic problems in relationships. Of the executive function tasks, the FAS verbal fluency test significantly correlated with pragmatic deficits. Despite executive function predicting a significant proportion of pragmatic variability, much variability was left unexplained, and the researchers concluded that this may potentially be attributed to ToM or other related deficits.

While the literature is currently divided in terms of identifying ToM or executive function difficulties as the primary cause of pragmatic deficits in individuals with TBIs, the most likely explanation involves a combination of many underlying and inter-related cognitive abilities. In an overview of the literature, Martin and McDonald (2003) stated how determining a single causal basis for pragmatic language may not be possible. Nonetheless, delineating some of the underlying processes causing pragmatic language impairment in individuals with TBI may be useful in identifying the most effective forms of treatment.

## INDIVIDUAL THERAPY FOR PRAGMATIC/SOCIAL COMMUNICATION

Few studies have focused on the remediation of social skills in individual therapy for TBI. Helffenstein and Wechsler (1982) described the effectiveness of Interpersonal Process Recall (IPR) in individual therapy. This study involved 16 participants between 17 and 35 years who had sustained a non-progressive brain injury. Participants were divided between therapy using IPR in sessions and non-therapeutic attention sessions. A total of 20 hours of therapy was conducted, and each session was one hour per day. A one month follow-up was also conducted. Pre- and post-test measurements were obtained through a variety of scales and inventories measuring social skill level, social self-concept, and social anxiety. In addition, two independent raters analyzed video-taped interactions between the participants and an unfamiliar staff member for demonstrations of empathy, attention, and genuineness among other aspects of communication. Upon identifying a deficient skill in the video, the participant and staff member developed, modeled, and rehearsed a more appropriate social behavior.

The study found participants had significantly improved self-concept in general and as it related to their interpersonal skills after IPR therapy. Other communication skills subjectively noted by observers were also improved. Despite the limitations of this study that included a relatively small number of participants, limited follow-up, and the intensive nature of this type of therapy, it is an example of a measurably successful individual pragmatic therapy program outside of a group setting.

Additionally, Radice-Neumann, Zupan, Tomita, and Willer (2009) compared the effectiveness of Facial Affect Recognition (FAR) training with stories involving emotional inference (SEI). Twenty-one participants with acquired brain injury including 19 participants with TBI were recruited. Eleven participants were randomly assigned to the FAR training group and 10 others assigned to the SEI group. The FAR training used a computer program with facial images of varying emotions and feedback for incorrect identification responses. Participants worked on describing how a variety of emotional events made them feel. In contrast, the SEI training focused on short stories incorporating contextual cues of characters’ emotions for analysis. Participants who received FAR training demonstrated improvement at recognizing facial emotions and making emotional inferences, and caregivers rated participants higher on their socioemotional behavior. SEI participants showed less improvement, but still made gains in emotional inferences post-therapy. The approach used by Radice-Newman et al. (2009) served as the basis for the current study.

Despite limited research into this type of pragmatic therapy, facial emotion recognition seems to be a potentially promising area for rehabilitation. Given the increasingly explored connection between ToM and pragmatics deficits in individuals with TBI, more studies in this area of pragmatics should be explored.

## TELEPRACTICE IN SPEECH-LANGUAGE PATHOLOGY

Relatively little research exists examining pragmatic therapy provided via telepractice. Nonetheless, telepractice or the delivery of services through real time telecommunication programs is becoming more common. This is especially evident in speech-language pathology where telepractice provides many benefits for clients living in remote areas (Mashima et al., 2003).

Specific to TBI telepractice intervention, Garcia-Molina et al. (2010) demonstrated the benefits of telerehabilitation for improving attention, memory, and executive function in 80 participants with TBI with moderate to severe deficits. A 10-week cognitive rehabilitation treatment program with five sessions a week was implemented. Forty participants received therapy in the hospital and the others received telerehabilitation services at off-site health centers. In both settings, participants received therapy through the PREVIRNEC telerehabilitation system which combines cognitive neuropsychological rehabilitation with customized individual therapy plans. A variety of neuropsychological cognitive assessments were administered pre- and post-therapy. Participants or their caregivers also filled out the Patient Competency Rating Scale (PCRS) pre- and post-therapy to measure the participants’ competence with daily life tasks. Overall, significant improvement was seen in both telerehabilitation groups’ cognitive performance and their competence with daily life tasks.

While telecommunication-based therapy is relatively new, a growing body of research supports this service delivery mechanism for improving a variety of cognitive-communication related concerns. It appears that individuals with TBI demonstrating benefits from in-person social communication therapy can also benefit from the same type of therapy conducted through telepractice.

The American Speech-Language-Hearing Association (ASHA, 2010) defines telepractice as “the application of telecommunications technology to deliver professional services at a distance by linking clinician to client, or clinician to clinician for assessment intervention, and/or consultation” (p.1). Although many synonymous terms are found in the literature (e.g., telehealth, telecommunication, and telerehabilitation), for purposes of this speech-language pathology study, the term telepractice will be used.

In summary, social communication therapy has generally produced positive results for individuals with post-acute and chronic TBI across a range of severity levels. Many studies have supported individual social communication therapy approaches such as Interpersonal Process Recall and Facial Affect Recognition. Therapy approaches targeting Theory of Mind and/or executive function deficits are well-documented in the literature, and telepractice appears to be an effective delivery system for various types of therapy.

Given the negative effects on social relationships post-TBI, largely due to pragmatic deficits, continued research in the area of social communication is necessary. Additionally, social communication therapy via telepractice is still a relatively unexplored area of research. Many individuals with TBI may live in remote areas where in-person social communication therapy is not readily accessible. For them, telepractice may be the most practical means to obtain services. While in-person FAR training has been found to be effective in improving the recognition of emotions, no study was found that examined the provision of FAR training via telepractice. Thus, a modified FAR training approach was used to assess participants’ ability to identify emotions via telepractice and to determine if skills generalized to overall social communication abilities. Therefore, the overall aims of the current research were:

1. To determine if a modified FAR training delivered via telepractice will influence facial affect recognition in participants with chronic (> 5 years) TBI based on pre- and post-evaluation using static pictures.
2. To determine if a modified FAR training delivered via telepractice will affect social communication abilities based on pre- and post-pragmatic measures as rated by unfamiliar observers.

## METHODS

### PARTICIPANTS

Volunteers were solicited for this study through brain injury support and training groups in Hawaii. Inclusionary criteria for adult participants in this study were: (1) moderate to severe TBI for initial Glasgow Coma Scale (GCS) score, (2) age 21–65 years, (3) at least five years post-injury, and (4) have sufficient cognitive and memory function to participate in telepractice therapy. Additionally, participants had to have a computer with Internet access, a web camera, and the ability to install a videoconferencing telecommunication program (i.e., VSee) for therapy sessions. The participants were required to complete telepractice evaluations related to emotional recognition and social communication before and after therapy. The participants were also required to have time in their weekly schedules to take part in one hour, bi-weekly telepractice therapy sessions for three weeks, and then continue with weekly, one hour therapy sessions for three weeks. Exclusionary criteria included: history of multiple TBIs, a history of severe depression or other psychiatric conditions, severe auditory or visual deficits, fine motor deficits, or other comorbid conditions that could affect the ability to participate in therapy via telepractice.

Two individuals voluntarily agreed to participate in the study. Participant one, a 53 year old male, had a moderate TBI (initial GCS of 12). He was 13 years post-injury and had been observed as having difficulty establishing and maintaining personal relationships, trouble reading facial expressions, and had challenges exhibiting appropriate social responses across a wide variety of everyday social situations. He was employed part-time.

Participant 2, a 44 year old male, had a severe TBI (initial GCS of <8). He was seven years post-injury and had been observed having difficulty initiating and maintaining conversations and trouble reading facial expressions. He was a part-time college student.

### MATERIALS

Items used in the study included consent forms for participants, and the VSee web-based teleconferencing program installed on personal computers (i.e., both PC and Mac) equipped with web cameras. The VSee telemedicine program allows for synchronous (real-time) interactions and reports to be HIPAA compliant (VSee, 2014). The program includes 720P high-definition video, FIPS 140-2 certified 256 bit AES encryption, and it is simple to download. VSee also allows multiple conversation partners (or observers) to participate in video conferences. By using the VSee program, conversations between the researcher and participants were compared pre- and post-intervention using the Pragmatic Protocol (Prutting & Kirchner, 1987). This protocol analyzes various categories within verbal, paralinguistic and nonverbal aspects of communication. Trained observers determined if participants were either *Appropriate* or *Inappropriate* in each category or indicated that they had *No Opportunity to Observe* a particular pragmatic behavior.

Additionally, a facial expression identification test was administered to participants pre- and post-telepractice therapy. The test included 2D facial emotional stimuli received from the University of Pennsylvania’s Brain Behavior Laboratory. This picture set was developed and standardized by Gur et al. (2002). The stimulus items included a wide variety of facial expression photographs across five emotional categories and two levels of intensity (e.g., happy, sad, anger, fear, no emotion; mild and extreme intensities).

During the modified Facial Affect Recognition (FAR) training, a set of 80 different facial expression emotion cards developed by Stages Learning was used. These cards covered a range of emotions including *happy*, *sad*, *angry*, *surprised* and *disgusted*. Pictures of additional emotions of facial expressions were also incorporated which included *uncomfortable*, *bored* and *neutral*. Lastly, stories featuring sarcasm in a dialogue were written by the primary researcher to teach the participant differences between facial expressions and actual spoken words.

### PROCEDURES

The study and consent form was approved by the Northern Arizona University (NAU) Institutional Review Board. During each research session, the primary researcher’s computer was connected through the NAU secure Internet server. Both the participant and primary researcher conducted therapy sessions in quiet, well-lit rooms. The research was completed in the NAU Department of Communication Sciences and Disorders, and both participants resided in Hawaii. The second researcher was certified and licensed to practice speech-language pathology in both AZ and HI, and supervised all research sessions.

Initially, the participants engaged in an hour-long baseline evaluation session via telepractice. The trained observers (two graduate students in speech-language pathology and a speech-language pathologist) in the current study learned about the Pragmatic Protocol (Prutting & Kirchner, 1987) in their graduate neurogenic courses, reviewed the categories related to the protocol, reviewed the Prutting and Kirchner (1987) article, and discussed any questions about items with the primary author. Specific examples and descriptions of various social communication behaviors were documented. The trained observers viewed each participant pre- and post-intervention during a semi-structured conversation with the primary researcher for 30 minutes and rated the interaction on the Pragmatic Protocol (Prutting & Kirchner, 1987). During this initial evaluation session, the participants were also presented with a set of 50 standardized photographs of people with five different emotions shown through facial expressions (Gur et al., 2002).

After the evaluation session, individual telepractice therapy occurred in 60-minute sessions for a period of six weeks focusing on a modified version of FAR training. Initially, sessions occurred twice a week for three weeks and then decreased to once a week for the remainder of the study. The second participant’s therapy did not start until the first participant was finished. Radice-Neumann et al. (2009) provide a detailed description of the facial affect recognition training which served as the basis for the therapy used in this study.

FAR training for this study was modified to fit the constraints of telepractice and the particular needs of the participants. All of the therapy was provided by the primary researcher. The first portion of the FAR training program used a systematic presentation of 2D images via a variety of static facial expression emotion cards varying in gender, intensity of emotion, and race. Two new emotions were presented each session along with a review of prior emotions. Emotions represented on the cards included: angry, sad, bored, thoughtful, happy, uncomfortable, fearful, and disgusted. For each picture card presented by the researcher, the participant was asked to identify the corresponding emotion. Initially, visual and verbal cues were used by the researcher to highlight relevant facial emotional features, but these were gradually eliminated over the course of therapy. Following incorrect identification of emotions, participants were given additional cues about various facial features in the pictures along with the correct emotion. For example, “The eyes are wide open, the eyebrows are raised, and the mouth is open. This person is fearful.” Participants were eventually asked to point out what facial features they had noticed instead of the researcher providing the verbal and visual cues. This portion of training continued for six sessions over three weeks.

The second part of the modified FAR training involved reflection by the participant upon the situations which corresponded to various emotions. Participants were asked to describe the physical and physiological changes that occurred in each of these situations and to give a detailed breakdown of each emotional event which included how the event made them feel and how they responded to the event. This second portion of the modified FAR training was conducted during one session.

A third part of the treatment was added to the FAR training to fit a particular difficulty that participants demonstrated with identifying sarcasm. Both researchers role-played various social stories characterized by sarcastic statements made within pre-written scripts. This portion of the therapy was conducted over two sessions. Participants were asked to identify if sarcasm was present in each situation, whether the characters’ statements matched their facial expressions, and what emotion the hypothetical characters were really feeling. During the final treatment session, participants also reviewed all of the emotions from the facial expression cards. Based on all the different tasks incorporated in the modified FAR training for this particular study, executive function and Theory of Mind were both addressed.

A final evaluation session occurred post-therapy. The participant and researcher were observed during a semi-structured conversation by the same three independent observers, and the interaction was again rated on the Pragmatic Protocol. Finally, the participant identified the emotion of 50 different facial expression cards from the same standardized set used in the initial evaluation.

### DESIGN AND DATA ANALYSES

Pre- and post-evaluation performance was presented for two case studies of adults with chronic moderate and severe traumatic brain injury (TBI). Baseline and final data percentages for the identification of correct facial affect using the 50 cards were collected. Inter-rater reliability analyses of the trained observers for scores on the Pragmatic Protocol were completed.

## RESULTS

### SPECIFIC AIM 1

To determine if a modified FAR training delivered via telepractice will influence facial affect recognition in participants with chronic (> 5 years) TBI based on pre- and post-evaluation using static pictures.

At baseline, Participant 1 identified 10/50 (20%) facial expression pictures correctly. The primary emotion identified accurately was happiness followed by anger. Participant 1 had difficulty identifying disgust, fear, and neutral expressions in the initial evaluation. During post-evaluation following therapy, Participant 1 identified 28/50 (56%) facial expression pictures correctly, representing a 36% increase in accuracy. Again, happiness and anger were commonly identified correctly, and the emotions of disgust and neutral facial expressions were identified more readily compared to the pre-assessment. The fearful expression was still difficult for the client to identify in the post-assessment.

At baseline, Participant 2 identified 24/50 (46%) facial expression pictures correctly. The primary emotion identified accurately most often was happiness followed by sadness. Participant 2 had relative difficulty identifying disgust and fearful expressions in the initial evaluation. During post-evaluation following therapy, Participant 2 identified 39/50 (78%) facial expression pictures, representing a 32% increase in accuracy. Again, happiness and sadness were most commonly identified correctly while the emotions of neutral, disgust, and fear were also identified more readily compared to the pre-assessment.

### SPECIFIC AIM 2

To determine if a modified FAR training delivered via telepractice will affect social communication abilities based on pre- and post-pragmatic measures as rated by unfamiliar observers.

Three independent observers watched a 30 minute interaction between the primary researcher and Participant 1. The Pragmatic Protocol (Prutting & Kirchner, 1987) was completed following the interaction. Items marked as *Appropriate* were scored as 2, *Inappropriate* scored as 1, and *No Opportunity to Observe* was scored as 0. Inter-rater reliability between the three trained observers was high, as total scores for pre- and post-scores were within +/− 5 points.

Another 30 minute interaction between the primary researcher and Participant 2 was reviewed by the trained observers. The inter-rater reliability between the observers was within +/− 39 points on total pre- and post-scores. Upon closer review of the items, the results of the protocols indicated that one of the observers found that five items (Topic Selection, Topic Introduction and Topic Change along with Physical Contact and Physical Proximity) in the verbal and non-verbal aspect sections could not be scored. Therefore, these items were rated as *No Opportunity to Observe* and received a score of zero. Inter-rater reliability between the other two observers was within +/− 10 points indicating more similar scoring of the protocol.

## DISCUSSION

The static emotion pictures used as pre- and post-measures in this study were developed by Gur et al. (2002). Each emotion clearly belonged to one of five emotional categories, and these pictures were entirely distinct from the facial expression cards used over the course of therapy. Results from Participant 1 showed a 36 percent increase pre- to post-therapy in the ability to determine emotions from the Gur et al. (2002) pictures. For Participant 2, there was a 32 percent increase. Based on the modified FAR training used in this study which included identifying emotions through static facial expressions, personally reflecting on those emotions, and identifying sarcasm and emotions within social stories and role-play, the increase in the participants’ percentages likely represent an improved understanding of emotions and emotional vocabulary.

The current modified FAR training incorporated both Theory of Mind (ToM) and executive functions, and the results indicated that even participants with chronic (> 5 years) TBI can show gains in pragmatic skills. Henry, Phillips, Crawford, Ietswaart, and Summers (2006) and Martin and McDonald (2003) all state that it is difficult to determine specifically whether executive functions, ToM, or a combination of both can account for all pragmatic skills. The modified FAR training required the use of both executive functions and ToM to generalize skills from static facial emotion cards to real-time, facial emotions used in role play. Additionally, the participants’ improvement in identifying emotions is consistent with Bornhofen and McDonald (2008), whose previous research showed improvement in participants with chronic TBI and their ability to identify emotions in faces. The current case studies also supports the findings from other studies (Garcia-Molina et al., 2010; Riegler, Neils-Strunjas, Boyce, Wade, & Scheifele, 2013) that have determined that telepractice is an effective method for providing services.

The Pragmatic Protocol (Prutting & Kirchner, 1987) was selected for this study due to its comprehensive rating of verbal, paralinguistic, and non-verbal aspects of communication. Findings in the current study showed high inter-rater reliability between observers for pragmatic skills pre- and post-therapy for Participant 1. Inter-rater reliability of observers for Participant 2 showed larger differences due to one observer’s identifying the lack of opportunity to view specific verbal and non-verbal aspects of communication. Braden et al. (2010) reported that the use of pragmatic rating scales that subjectively measured social communication abilities seldom found statistically significant differences. After review of the scores and items of the Pragmatic Protocol, the authors determined that this particular measure may not have fully captured the emotional component of the modified FAR training program, particularly due to the nature of the conversation that was observed. Few spontaneous emotions were produced during the interaction evaluated by the observers. This may have been typical of males and their conversational styles during interactions.

Several limitations were evident in the present study. One limitation of the current study was the small number of participants. A larger sample size may have added support for the current therapy provided via telepractice and determined if the findings could be generalized to the chronic, moderate to severe TBI population. Future research with additional participants is necessary to determine if the modified FAR training via telepractice is truly effective.

As previously mentioned, another limitation may have been the communication measure used for this study. The Prutting and Kirchner Pragmatic Protocol did not focus specifically on the emotional component of the FAR training program. Additional measures specifically evaluating gains in emotional awareness and how participants respond to emotionally charged situations may provide a more comprehensive picture of the modified FAR training program’s success in future studies. Additionally, the researcher and both participants were males. Perhaps, more pragmatic deficits would have been observed if the genders were different.

A final limitation of this study was the use of web-based synchronous (real-time) devices for telepractice. Many participants with TBI who wanted to take part in the study did not have access to a computer with a camera for telepractice purposes. This may have been due to finances, the cost of Internet services, safety issues and/or problem solving difficulties. Over the course of therapy, several sessions with participants also had to be delayed due to scheduling issues or discontinued due to slow Internet connections. These difficulties were resolved by rescheduling appointments with the participants, but may present additional challenges for many individuals with TBI.

## CONCLUSION

This study investigated a telepractice-based therapy intervention designed to improve facial affect recognition and overall social communication skills in two participants with chronic traumatic brain injury. Overall, the therapy program that was developed showed positive changes in the ability of participants to understand facial expressions/emotions. The lack of pragmatic changes identified in this study may suggest that identifying emotions on facial expressions is too specific for pragmatic tools to measure and is only one component of social communication. Certainly, there is a need for continued research in this area.

The case studies supported the feasibility of this type of therapy administered through telepractice. Implementation of FAR training was manageable through telepractice with minimal cost to the participants who could download the appropriate telecommunication software program. While this study had volunteer participants over the age 40 due to their ability to provide the time necessary for this project, younger participants may have increased access to personal computers, laptops, and tablet devices with real-time interaction programs and capabilities. This should be considered when targeting populations for future telepractice-based studies.

Additionally, the increase in the participants’ accuracy in identifying emotions from static facial expression pictures pre- to post-assessment likely represents an increase in the understanding of emotions and emotional vocabulary. Perhaps the increased knowledge of emotionally-related words allowed the participants with TBI to discuss their feelings and understand the feelings of others in their everyday life.

The ability to read facial expressions remains an important part of communication, specifically Theory of Mind, and should not be neglected in speech-language therapy. Ultimately, telepractice-based therapy holds great potential for individuals with TBI who have difficulty accessing social communication therapy services. Those living in more remote locations that may lack the opportunity to obtain services and practice the use of pragmatic skills can do so with telepractice.
